# Automated Segmentation of Fetal Intracranial Volume in Three‐Dimensional Ultrasound Using Deep Learning: Identifying Sex Differences in Prenatal Brain Development

**DOI:** 10.1002/hbm.70058

**Published:** 2024-12-04

**Authors:** Sonja M. C. de Zwarte, Jalmar Teeuw, Jiaojiao He, Mireille N. Bekker, Ruud J. G. van Sloun, Hilleke E. Hulshoff Pol

**Affiliations:** ^1^ Department of Psychiatry, UMC Brain Center University Medical Center Utrecht, Utrecht University Utrecht The Netherlands; ^2^ Department of Developmental Psychology Utrecht University Utrecht The Netherlands; ^3^ Department of Experimental Psychology, Helmholtz Institute Utrecht University Utrecht The Netherlands; ^4^ Department of Obstetrics University Medical Center Utrecht Utrecht The Netherlands; ^5^ Lab of Biomedical Diagnostics, Department of Electrical Engineering Eindhoven University of Technology Eindhoven The Netherlands

**Keywords:** 3D ultrasound, automated segmentation, convolutional neural network, fetal brain development, intracranial volume, sex differences

## Abstract

The human brain undergoes major developmental changes during pregnancy. Three‐dimensional (3D) ultrasound images allow for the opportunity to investigate typical prenatal brain development on a large scale. Transabdominal ultrasound can be challenging due to the small fetal brain and its movement, as well as multiple sweeps that may not yield high‐quality images, especially when brain structures are unclear. By applying the latest developments in artificial intelligence for automated image processing allowing automated training of brain anatomy in these images retrieving reliable quantitative brain measurements becomes possible at a large scale. Here, we developed a convolutional neural network (CNN) model for automated segmentation of fetal intracranial volume (ICV) from 3D ultrasound. We applied the trained model in a large longitudinal population sample from the YOUth Baby and Child cohort measured at 20‐ and 30‐week of gestational age to investigate biological sex differences in fetal ICV as a proof‐of‐principle and validation for our automated method (*N* = 2235 individuals with 43492 ultrasounds). A total of 168 annotated, randomly selected, good quality 3D ultrasound whole‐brain images were included to train a 3D CNN for automated fetal ICV segmentation. A data augmentation strategy provided physical variation to train the network. K‐fold cross‐validation and Bayesian optimization were used for network selection and the ensemble‐based system combined multiple networks to form the final ensemble network. The final ensemble network produced consistent and high‐quality segmentations of ICV (Dice Similarity Coefficient (DSC) > 0.93, Hausdorff Distance (HD): HD_voxel_ < 4.6 voxels, and HD_physical_ < 1.4 mm). In addition, we developed an automated quality control procedure to include the ultrasound scans that successfully predicted ICV from all 43492 3D ultrasounds available in all individuals, no longer requiring manual selection of the best scan for analysis. Our trained model automatically retrieved ultrasounds with brain data and estimated ICV and ICV growth in 7672 (18%) of ultrasounds in 1762 participants that passed the automatic quality control procedure. Boys had significantly larger ICV at 20‐weeks (81.7 ± 0.4 mL vs. 80.8 ± 0.5 mL; B = 2.86; *p* = 5.7e‐14) and 30‐weeks (257.0 ± 0.9 mL vs. 245.1 ± 0.9 mL; B = 12.35; *p* = 8.2e‐27) of pregnancy, and more pronounced ICV growth than girls (delta growth 0.12 mL/day; *p* = 1.8e‐5). Our automated artificial intelligence approach provides an opportunity to investigate fetal brain development on a much larger scale and to answer fundamental questions related to prenatal brain development.

## Graphical abstract

A convolutional neural network model was developed to automatically segment fetal intracranial volume (ICV) from 43492 three‐dimensional ultrasounds. We developed an automated quality control procedure, no longer requiring manual selection. As proof‐of‐principle, we showed that boys have larger ICV at 20‐ and 30‐weeks of pregnancy, and ICV‐growth than girls (*n* = 1762).

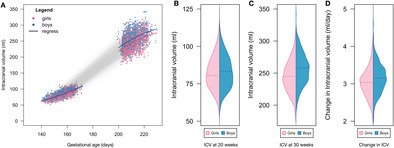

## 1 Introduction

Ultrasound imaging is widely used to monitor fetal growth and development during pregnancy due to its low‐cost, non‐invasive, and real‐time characteristics. The developing fetal brain can also inform on functional outcomes postnatally (Davies et al. 2020; Gao et al. 2019; Clifford et al. 2016; Hulshoff Pol et al. 2000; Gillman 2005; Schlotz and Phillips 2009; Norris et al. 2018; Henrichs et al. 2010). It has been shown that a more pronounced fetal brain size and growth in early‐ to mid‐gestation was positively associated with academic attainment in mid‐childhood learning capacity in the area of mathematics, writing, reading, and logical thinking (Norris et al. 2018). Moreover, a faster growth from mid to late pregnancy predicted a lower risk of delayed infant development at 12 months of age (Henrichs et al. 2010). In addition, a large and steadily accumulating body of evidence suggests that abnormal prenatal brain development is associated with several psychiatric disorders, such as in psychosis (Davies et al. 2020).

Most anatomical knowledge on fetal brain development comes from post‐mortem studies (Chi, Dooling, and Gilles 1977), providing highly detailed reference atlases (e.g., https://atlas.brain‐map.org/), and exceptional work on gene expression and cell composition. However, post‐mortem studies have several limitations when studying typical brain development: e.g., the inherently cross‐sectional design, deformation of the brain after extraction, and post‐mortem cases typically represent cases with abnormalities. In recent years, magnetic resonance imaging (MRI) studies have provided deeper insights into the structural and functional aspects of fetal brain development (Andescavage et al. 2017; Turk et al. 2019; Khan et al. 2019). While providing much greater contrast than ultrasound, fetal MRI studies investigating typical brain development are generally limited by small sample sizes and lack longitudinal measurements in utero. Longitudinal 3D ultrasounds can vastly expand our knowledge on typical human fetal brain development and how fetal brain structure and growth is related to outcome later in life remains limited.

Accurate automated image segmentation is a prerequisite for the quantitative assessment of the fetal brain in large‐scale ultrasound studies. Recent work has demonstrated the remarkable performance of deep learning techniques on automated biometrics measurement and segmentation of brain structures in both 2D and 3D ultrasound images (Moser et al. 2020; Simonyan and Zisserman 2014; Sinclair et al. 2018a, 2018b; Ronneberger, Fischer, and Brox 2015; Van Sloun et al. 2021; Namburete et al. 2018, 2023; Hesse et al. 2022; Cerrolaza et al. 2018; Perez–Gonzalez et al. 2020; Yeung et al. 2021). Deep learning is a type of machine learning and artificial intelligence which processes data using multiple layers of complex structures or multiple processing layers composed of multiple nonlinear transformations, simulating the human neural network. By combining multiple nonlinear processing layers, the original data is abstracted layer by layer, and different levels of abstract features are obtained from the data and used for target detection, classification or segmentation. The advantage of deep learning is to replace the manual acquisition feature with unsupervised or semi‐supervised feature learning and hierarchical feature extraction efficient algorithms. Moreover, measurements generated in near real time by deep learning techniques have the potential to speed up clinical workflow. However, processing of fetal ultrasound images for quantification poses additional challenges. For instance, during routine ultrasound examinations (often) several images are acquired of the whole brain or focusing on specific fetal (brain or other) anatomy, with varying degrees of fetal movement. Selection of the best ultrasound for quantification requires training, takes considerable time, is subject to bias, and results in considerable loss of potentially relevant data.

Here, the aim of our study was twofold. One, we built a neural network for automated fetal brain extraction and ICV measurement from 3D ultrasound images acquired at 20 and 30 weeks of gestation. We performed a data augmentation and k‐fold cross validation with the Bayesian Optimization strategy. We have built upon previous work (Caspi et al. 2022), where we have employed an atlas‐based registration procedure for fetal brain annotations. While being successful at quantifying the fetal brain from 3D ultrasound, we were restricted, for example, by fetal pose and fetal age, and therefore unable to completely automate the segmentation procedure, highlighting the need to implement a deep learning rather than a more classical approach. Two, the three optimal networks were combined as an ensemble network to achieve a robust automated segmentation of fetal ICV at both 20 and 30 weeks of gestation without prior selection of ultrasound material that included brain information. In addition, we developed an automated quality control procedure to deal with many within‐subject images. In a large longitudinal population cohort, the model was applied to investigate whether the model predicted ICV by investigating sex differences in fetal ICV development.

## 2 Materials & Methods

### 2.1 Study Sample and Image Acquisition

The 3D ultrasound scans were obtained from the YOUth Baby and Child cohort study (https://www.uu.nl/en/research/youth‐cohort‐study) (Onland‐Moret et al. 2020). In this cohort, over 2800 typically developing children from the greater Utrecht region in the Netherlands are being followed from the fetal stage to childhood. As YOUth aimed to investigate the range of typical behavioral development, all pregnant women that were willing to participate were included (Onland‐Moret et al. 2020). After birth, children were only excluded [from longitudinal follow‐up assessments] if they were not mentally or physically capable of performing the tests during the visits to our center. Moreover, at longer‐term followup at the appropriate age, all participants had to master the Dutch language sufficiently to be able to understand all information and instructions. In case of a twin pregnancy, only one child per twin pair was included. The choice of which child would participate was made by the parents. Sex was determined after birth, categorizing the children as either female or male based on primary physical characteristics. We acknowledge that a binary definition of the sexes is limited and falls short in describing the rich variation in sexes that is present (Heidari et al. 2016). However, at such a young age, the rich variation in sex and gender differences is not easily detectable.

During pregnancy, transabdominal ultrasounds were acquired at two time points, for example, around 20 and 30 weeks of gestation. The sweeps were made by experienced sonographers with a Voluson E10 machine (GE Healthcare, Zipf, Austria), who also performed routine prenatal ultrasound assessments during the visits. To acquire a 3D ultrasound image, the probe sends out a sweep with sound waves at different angles, and the returning echoes are processed and reconstructed in a multiplanar view of the three two‐dimensional (2D) orthogonal planes (Albers et al. 2018). In this study, ultrasound scans from 2235 participants were included. On average, each participant had 10 ultrasound sweeps per measurement, resulting in over 43000 ultrasound images of various fetal anatomy and quality. At both visits, the protocol included at both visits five volume sweeps of the fetal brain from each participant. Two volumes were acquired from the axial plane (transthalamic and transcerebellar), two from the coronal plane (transthalamic and transcerebellar), and one from the midsagittal plane (Albers et al. 2018). Therefore, in each individual fetus, on average at least half of the ultrasounds covered most of the whole brain.

### 2.2 Image Preprocessing

All 43492 ultrasound volumes were converted to NIFTI (4D View, GE Healthcare, Zipf, Austria). An affine registration (12° of freedom), to account for variation in anatomy and image acquisition, was used to align the ultrasound volumes to a gestational age‐specific ultrasound template of the fetal brain with FSL FLIRT (Jenkinson and Smith 2021; Jenkinson et al. 2002). Only the rigid transformation (6° of freedom; for example, only translation and rotation) of the affine registration was applied to the original ultrasound volume to orient the fetal brains in roughly the same orientation as the template, after which the volumes were cropped automatically to retain only the heads of the fetus and resampled to a fixed isotropic voxel size of 0.40 mm.

### 2.3 Network Pipeline

A total of 168 high‐quality 3D ultrasound images of typical developing fetal brains with corresponding ICV annotation were included for the development of the neural network to automatically segment fetal ICV. These images were part of the first internal data release of the YOUth Baby & Child cohort consisting of about 200 scans from the pool of ongoing data collection, and together with their annotation, passed manual quality inspection (Caspi et al. 2022; Albers et al. 2018). Quality control of the ultrasound images and annotations was performed by experts trained to assess fetal ultrasound scans. In total, 95 subjects (41 female, 53 male, 1 unknown) were included: 73 subjects with both a 20‐ and 30‐week ultrasound, 14 subjects with only a 20‐week ultrasound, and 8 subjects with only a 30‐week ultrasound. The quality of the ultrasound scan and/or annotation was deemed insufficient for training in the remaining cases. The 168 ultrasound images were split into a training dataset (total N_train_ = 116, evenly distributed across both age groups) and a test dataset (N_test_ = 52; N_test 20w_ = 29, N_test 30w_ = 23). For sample characteristics, see Table S1. To prevent bias, we ensured that subjects with two scans were not both part of the train and test data.

#### 2.3.1 Data Augmentation

The network relies on manual annotations for accurate learning. However, manually annotating fetal brain structure from 3D US images is challenging and time‐consuming because of fetal movement, low resolution, and artifacts caused by maternal tissue. Therefore, we only have limited data available to train the network. To overcome this issue, we applied a data augmentation strategy which is a common approach to create more data and to adding physical variations of the fetal brain imaging (e.g., brain size, location, orientation, and quality of imaging) which increased the diversity of our data (Van Sloun et al. 2021; Shorten and Khoshgoftaar 2019). Even with an enlarging sample size, it is still beneficial to include data augmentation acting as a regularizer and helping reduce overfitting when training a machine learning model (Shorten and Khoshgoftaar 2019). The train data was augmented three times, which increased the number of images as well as annotations by random spatial augmentations (elastic, rotation, scaling, mirroring deformation), color augmentations (contrast, brightness, gamma transformation), and noise augmentations (Gaussian noise addition, gaussian blurring transformation) (Figure S1) (Isensee et al. 2020).

After data augmentation, the training dataset included 464 3D images. All data, including the original data (*N* = 112) and the augmented data (*N* = 3*112 = 336), were resized to 128×128×128 voxels, and then each color channel of one voxel of 3D images was normalized to numeric values from 0 to 1 while that of 3D annotation was normalized and converted to binary values, that is, 0 or 1.

#### 2.3.2 Convolutional Neural Network Architecture

**FIGURE 1:**
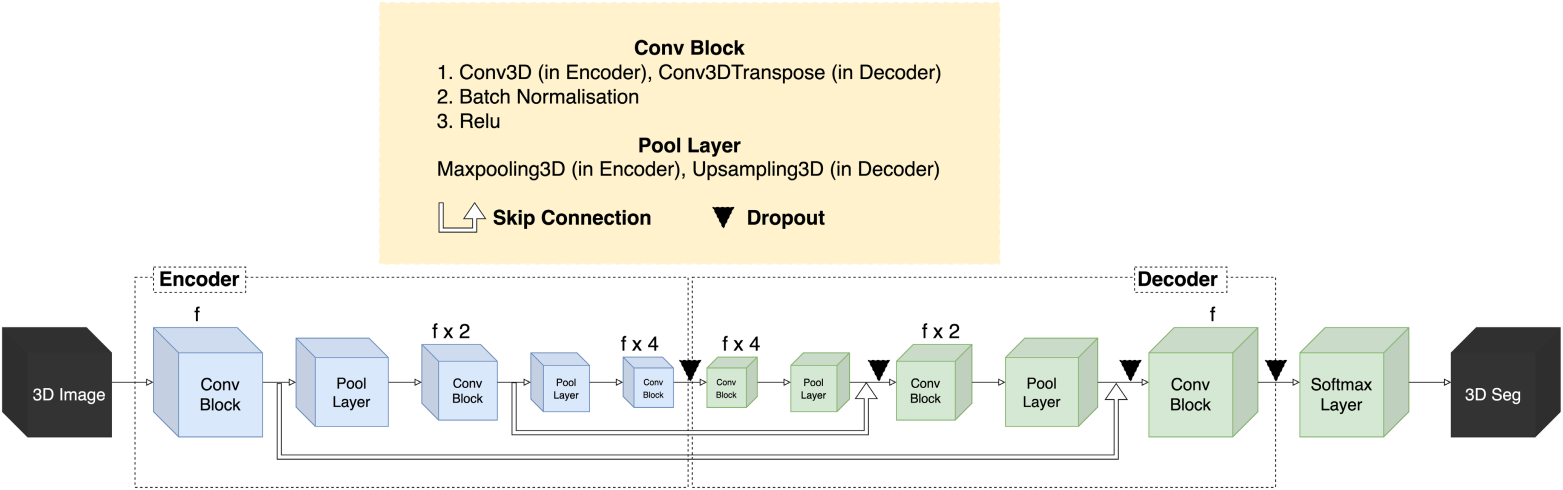
The structure of the 3D convolutional neural network. The convolutional (Conv) block starts with one pre‐defined filter size (*f*) and then a multiple of it in the flow from 3D images to 3D segmentations. The kernel size inside the convolution layer is 5 or 3 and the pooling size of the sampling layer is 2.

A 3D convolutional neural network (CNN) was trained for the automated segmentation of fetal ICV. The CNN architecture comprises an encoder‐decoder network with skip concatenation between them. The architecture contains the convolutional block with batch normalizations and ReLu activations, downsampling/upsampling layers, dropout layers, and a softmax layer before output. The convolutional block starts with one pre‐defined filter size and then a multiple of it in the flow. The kernel size inside the convolution layer is 5 or 3 and the pooling size of the sampling layer is 2 (Figure 1). The network was trained with Adam optimizer, Sparse Categorical Cross‐Entropy loss function, and two metrics, that is, voxel‐wise accuracy and Dice Similarity Coefficient (DSC).

#### 2.3.3 Network Selection

Next, we performed 3‐fold cross‐validation and Bayesian Optimization based on the DSC of the validation dataset to tune the network hyperparameters for batch size, filter size and learning rate values. The pre‐defined filter size ranged from 8 to 64, batch size from 16 to 64 and learning rate from 0.0001 to 0.01. The hyperparameters were set to be optimized for eight iterations by randomly exploring and adjusting parameters based on the previous random points, as well as simultaneously conducting the 3‐fold cross‐validation scheme, resulting in 24 networks. Each network performs the same goal of segmenting the ICV in ultrasound images, however, varies in performance because of different (hyper) parameters. We used 3‐fold cross‐validation and ensemble learning to ensure stability and generalizability of the performance of the trained model on independent datasets. For each fold, voxel‐wise accuracy and DSC were calculated during training, and Wilcoxon signed‐rank tests were performed to identify the best performing network for each fold with statistical significance. These three networks were combined as an ensemble network by a voting‐based technique (i.e., for each voxel the predicted label is assigned by the value with the most votes from the sub‐networks) (Polikar 2006; Kotu and Deshpande 2014).

#### 2.3.4 Performance Assessment

ICV masks were predicted by the ensemble network, and assessed by four metrics (voxel‐wise accuracy, DSC, Hausdorff Distance voxel distance (HD_voxel_), and Hausdorff Distance physical distance in mm (HD_physical_)). High DSC and voxel‐wise accuracies represent a high level of similarity between the predicted and ground truth masks, meaning that the segmentation model or algorithm is performing well, whereas HD is a widely used performance measure to calculate the distance between two point sets, with low HDs representing good overall performance. Wilcoxon rank‐sum tests determined whether the ensemble network performed differently for the 20‐ and 30‐week age groups. All statistical tests considered *p* < 0.05 as significant. Each group per metric distribution except HD_physical_ was checked for outliers as the relative contour distance under the same voxel volume was more valuable to consider than the absolute distance under different physical volumes.

#### 2.3.5 ICV Measurement

Next, the model was applied to all reoriented ultrasound volumes to predict the ICV mask. ICV (*V*) was calculated by directly counting the voxels of the predicted segmentation in the ensemble mask (*N*), multiplied by the voxel size (*S*; in milliliter [ml]):

V=N*S

### 2.4 Automated Quality Control Procedure

An automated quality control procedure was performed after prediction of ICV in each ultrasound scan. Two gestational‐age specific ultrasound templates and corresponding ICV masks were constructed based on images from the annotated dataset (Caspi et al. 2022). The ultrasound images were aligned to the appropriate template through affine registration with FSL FLIRT (Jenkinson and Smith 2021; Jenkinson et al. 2002), and the template ICV mask was brought back into native/individual space through inverse of the affine transformation. The DSC between the predicted ICV mask and transformed template ICV mask was computed. High DSC scores (approaching 1.0) indicated high agreement between the two types of ICV mask, whereas low DSC (approaching 0.0) indicated low agreement.

We validated the automated DSC QC score by randomly selecting 2040 ultrasounds and predicted ICV masks for manual inspection. A single expert rated the ICV masks overlayed on their ultrasound to assess the quality of the segmentation. The rater was blind to the estimated DSC QC score. ICV segmentations with a DSC QC score of 0.90 to 1.00 were almost all rated “Great” or “Okay” (Figure S2A). Starting at the bin with DSC QC scores between 0.85 and 0.90, only ~67% of the segmentations were considered “Great” or “Okay”, with the percentage dropping rapidly for lower DSC QC scores. Another view of this data shows the same trend of decreasing average DSC QC score with lower quality ratings by the expert (Figure S2B). We observe a gradual drop‐off in the number of ultrasound scans with increasing higher scores for DSC QC, with the number of subjects with at least one ultrasound remaining steady until about a DSC QC score of 0.80 (Figure S3). For this proof‐of‐application investigation into sex effects in the brain during prenatal development, we decided on a more conservative DSC QC score threshold of > 0.90, retaining about 23% of all ultrasounds, and 90% of all subjects, for which the overwhelming majority of segmentations are considered “Great” or “Okay.”

### 2.5 Statistics

All statistical analyses were performed using *R* (http://www.r‐project.org) on the sample that passed automated quality control (*N* = 7672 images from *N* = 1762 individuals; for sample characteristics, see Table S2). Linear regression analysis of the intracranial volumes was performed with the nlme package in R, at each wave separately (http://CRAN.R‐project.org/package=nlme) (Pinheiro and Bates 2000). The models included fixed effects for sex, linear age, quadratic age, and random intercepts for each subject to account for the repeated measures both within and between longitudinal assessments.

Longitudinal change rates in intracranial volume were computed as the linear difference between the intracranial volume at 30 weeks follow‐up and 20 weeks baseline for the ICV masks with the highest DSC quality score (*N* = 849 individuals with longitudinal ultrasounds that passed automated quality control; Table 1). The change rates were converted to daily change rates to account for variation in sampling interval between longitudinal assessments. Daily linear change rates were compared between sexes with a Welch's t‐test.

**TABLE 1:** Demographics table and fetal intracranial volume (ICV).

Measure	Sample	Total	Girls	Boys	Difference between sexes
Participants (N)^a^	All	1762	863 (49.0%)	899 (51.0%)	χ^2^ = 1.391, df = 1, p‐value = 0.238 [n.s.]
	20 weeks	1231	646 (47.5%)	585 (52.5%)	χ^2^ = 5.849, df = 1, p‐value = **0.016**
	30 weeks	1380	710 (51.4%)	670 (48.6%)	χ^2^ = 2.204, df = 1, p‐value = 0.067 [n.s.]
	Longitudinal	849	432 (50.9%)	417 (49.1%)	χ^2^ = 0.462, df = 1, p‐value = 0.497 [n.s.]
Ultrasounds (N)^a^	20 weeks	4096	1949 (47.6%)	2147 (52.4%)	χ^2^ = 18.950, df = 1, p‐value = **1.3e‐5**
	30 weeks	3576	1858 (52.0%)	1718 (48.0%)	χ^2^ = 10.806, df = 1, p‐value = **0.001**
	Longitudinal	1698	864 (50.9%)	834 (49.1%)	χ^2^ = 0.991, df = 1, p‐value = 0.320 [n.s.]
Age (days)^b^	20 weeks	155.0 ± 6.2 [140; 171]	155.7 ± 6.2 [141; 171]	154.4 ± 6.2 [140; 171]	*t* = 3.738, df = 1217.7, p‐value = **1.9e‐4**
	30 weeks	211.5 ± 5.7 [199; 230]	211.6 ± 5.7 [201; 230]	211.4 ± 5.8 [199; 229]	t = 0.478, df = 1368.5, p‐value = 0.633 [n.s.]
	Interval	56.7 ± 7.8 [37; 81]	56.2 ± 7.6 [38; 79]	57.1 ± 8.0 [37, 81]	*t* = −1.636, df = 842.0, p‐value = 0.102 [n.s.]
Uncorrected ICV (ml)^c^	20 weeks	81.3 ± 0.3 [51.2; 124.0]	80.8 ± 0.5 [53.4; 124.0]	81.7 ± 0.4 [51.2; 118.4]	χ^2^ = 1.862, df = 1, p‐value = 0.172 [n.s.]
	30 weeks	250.9 ± 0.7 [176.7; 341.8]	245.1 ± 0.9 [176.7; 317.3]	257.0 ± 0.9 [185.1; 341.8]	χ^2^ = 81.223, df = 1, p‐value = **2.0e‐19**
Uncorrected change in ICV (ml)^d^	Longitudinal	174.0 ± 26.2 [97.6; 269.4]	169.6 ± 25.7 [97.6; 242.0]	178.6 ± 26.0 [110.8; 269.4]	*t* = −5.047, df = 845.27, p‐value = **5.5e‐7**
Uncorrected change in ICV (ml/day)^d^	Longitudinal	3.09 ± 0.39 [2.02; 4.64]	3.03 ± 0.39 [2.05; 4.23]	3.15 ± 0.40 [2.02; 4.64]	t = −4.317, df = 843.81, p‐value = **1.8e‐5**

^a^ Differences between sexes for number of participants and total number of ultrasounds included in the study were tested with a two‐proportions Z‐test testing for equal proportions.

^b^ The mean, standard deviation, and range [minimum; maximum] are reported for gestational age in days. Differences between sexes were tested with a two‐tailed Welch's t‐test testing for equal means.

^c^ The regression coefficient and its standard error, and range [minimum; maximum] are reported for the uncorrected ICV in milliliter (ml) after accounting for non‐independence between repeated measurements from the same individuals at each 20 and 30 weeks. Differences between sexes were tested with a log likelihood ratio test of the model with a null model excluding sex as independent variable.

^d^ The mean, standard deviation, and range [minimum; maximum] are reported for uncorrected change in ICV in ml. Differences between sexes were tested with a two‐tailed Welch's t‐test testing for equal means.

## 3 Results

### 3.1 Model Construction

#### 3.1.1 Hyperparameter Optimization and Cross‐Validation

**FIGURE 2:**
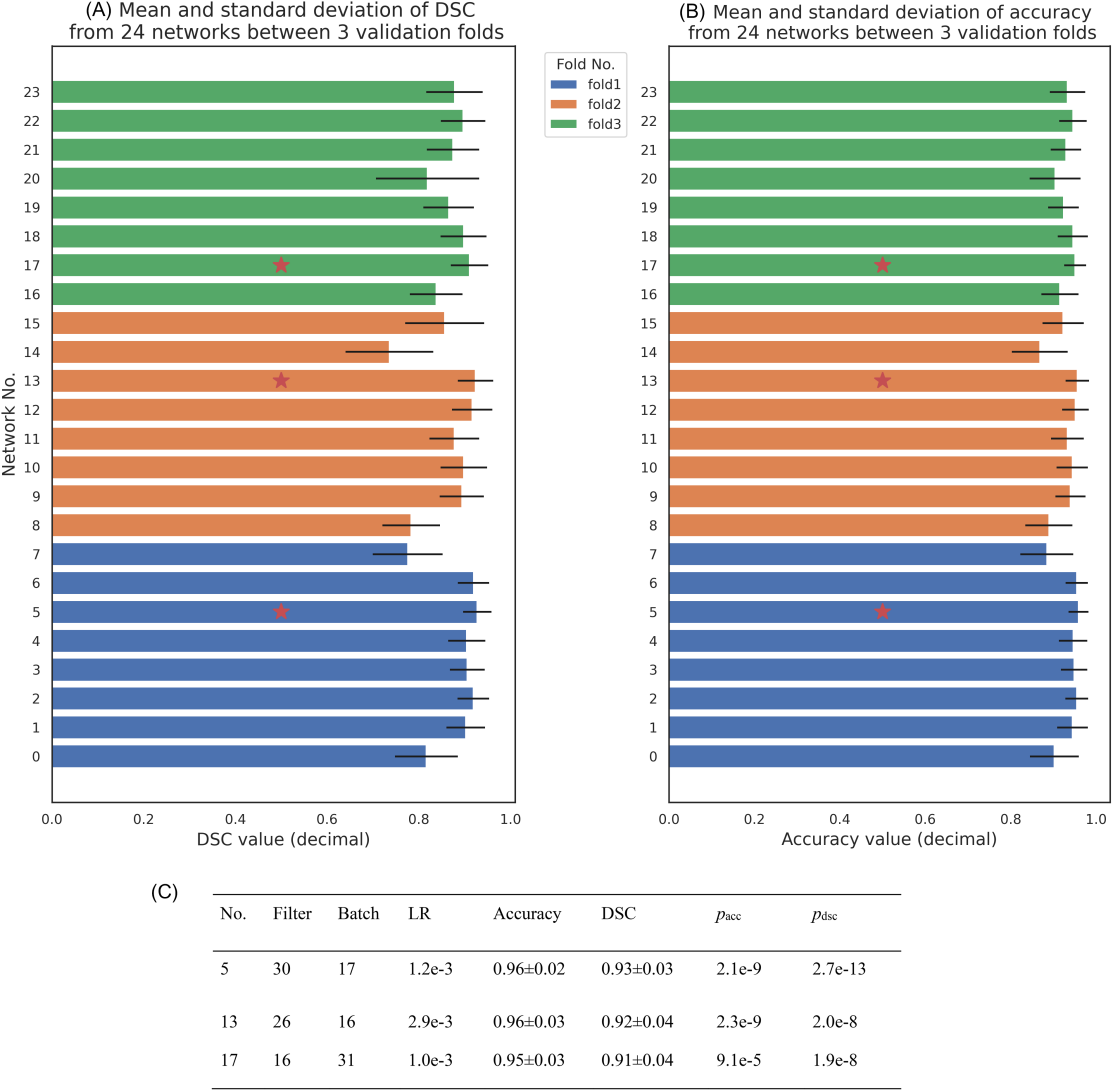
The performance during training of the 24 networks. The mean and standard deviation of (A) dice similarity coefficient (DSC) and (B) voxel‐wise accuracy on validation data in each of the 24 networks are depicted by the bars and lines respectively. Eight networks were grouped per cross validation: Fold 1 (blue), fold 2 (orange) and fold 3 (green). The red star markers refer to the best performance per metric per fold. (C) The hyperparameters and performance of the best three networks of each fold. The filter size, batch size and learning rate (LR) of networks No. 5, 13 and 17 are in (16, 32), (16, 32) and [0.001, 0.005) respectively. The *p*‐values of voxel‐wise accuracy (*p*_acc_), and (DSC) (*p*_dsc_), indicate the maximum values for the comparison of three networks to other networks in the same cross‐validation fold determined by the Wilcoxon signed‐rank test.

The 24 networks were trained across three validation folds with different ranges of hyperparameters for filter size (full range [8; 64]), batch size (full range [16; 64]), and learning rate (full range [0.0001; 0.0100]) to find the optimal network configuration. Trial runs overwhelmingly indicated a filter size range of (16, 32), a batch size of (16, 32), and learning rate of (0.0010; 0.0050) as optimal ranges with high voxel‐wise accuracy and spatial overlap (Table S3). The best performing networks for each validation fold were, No. 5, 13, and 17, were selected for their highest score on the mean of the spatial overlap of the predicted and observed mask (dice similarity coefficient; DSC) and accuracy of the predicted masks (Figure 2, and Figure S4). These three networks were combined into the final ensemble network where majority voting is used to determine the inclusion of a voxel in the ICV mask. This ensemble network was used to predict ICV in the larger YOUth sample.

#### 3.1.2 Model Testing

**FIGURE 3:**
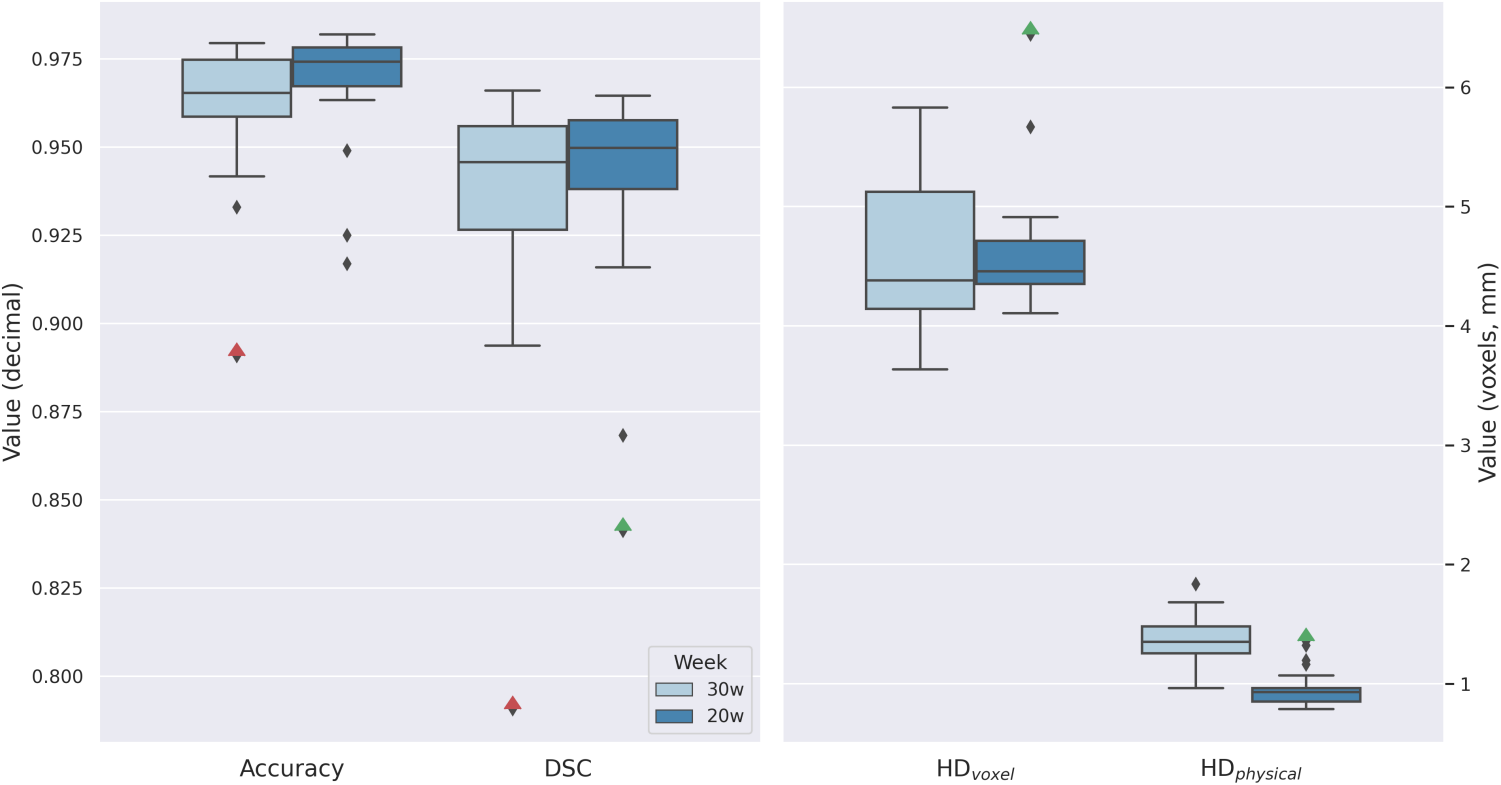
The distribution of 20‐week (dark blue) and 30‐week (light blue) test data for four metrics, that is, voxel‐wise accuracy and dice similarity coefficient (DSC), and the right y‐axis indicate the scale of Hausdorff Distance in voxels (HD_voxel_) and physical HD in millimeters (HD_physical_). The red and green triangles represent two recurring outliers.

The ICV predictions showed highly overlapping results as compared to the existing ICV annotations in both the 20‐ and 30‐week age groups, with respectively, voxel‐wise accuracy of 0.97 and 0.96, DSC 0.94 and 0.93, HD_voxel_ 4.59 and 4.56 voxels, HD_physical_ 0.95 and 1.35 mm (Figure 3). There was no significant difference in performance between the 20‐ and 30‐week test data based on DSC (*p* = 0.33) and HD_voxel_ (*p* = 0.49), while the voxel‐wise accuracy did show a significant difference in performance between the 20‐ and 30‐week age group (*p* = 0.03).

### 3.2 Sex Differences in ICV Development

Our model predicted ICV measures in *N* = 7672 ultrasound images from 1762 fetuses (Table 1; Table S2). This was 18% of the available ultrasound scans and 72% of the participants. Most unsuccessful predictions were due to non‐brain ultrasound, low‐quality ultrasound (e.g., due to motion of fetus or high maternal BMI), or incomplete coverage of the entire fetal brain. See Figure S5 for examples of ICV masks overlayed on top of corresponding ultrasound and DSC QC score.

**FIGURE 4:**
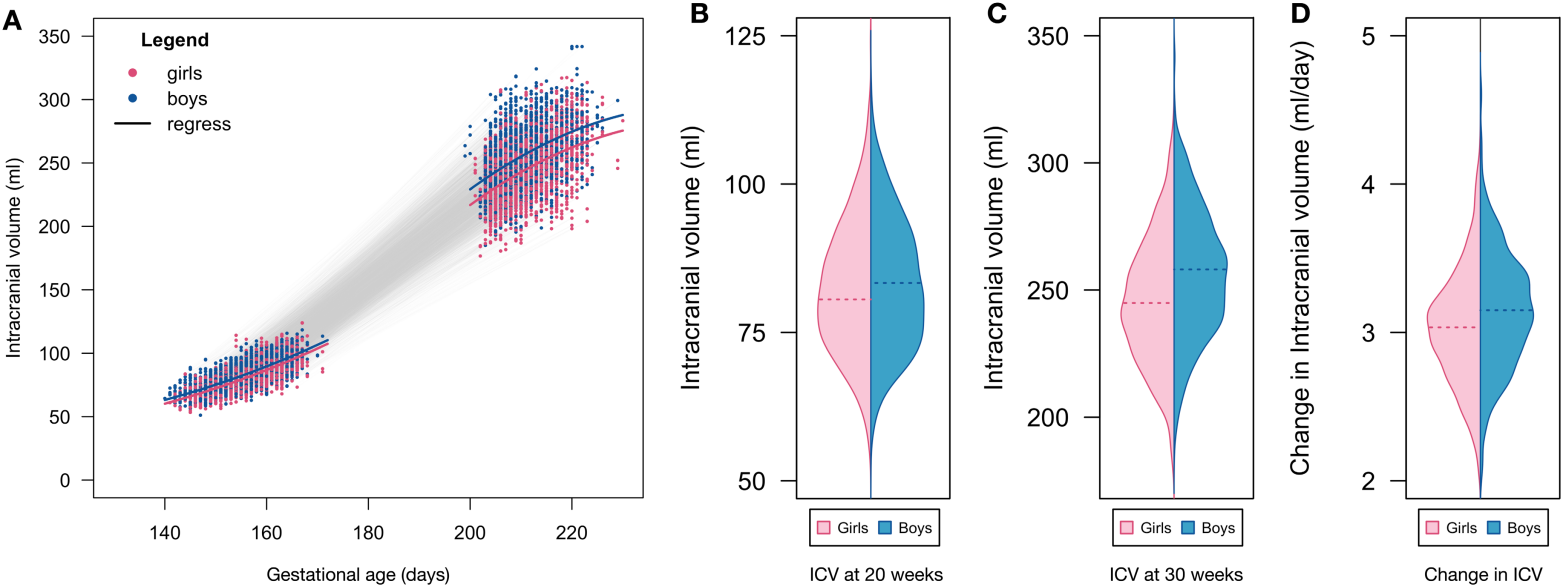
Growth curve and sex effects for fetal ICV of boys and girls. (A) Growth curve of fetal ICV at 20 and 20 weeks. Models included a quadratic age effect and sex effect. (B, C) Sex effect for fetal ICV of boys and girls at baseline 20 weeks and follow‐up 30 weeks. Sex effects were significant at baseline 20 weeks (*B* = 2.86 ± 0.38 (s.e.); *p* = 5.7e‐14) and follow‐up 30 weeks (*B* = 12.35 ± 1.13 (s.e.); *p* = 8.2e‐27) after correcting for linear and quadratic age effects. (D) Daily linear growth rates for boys and girls between baseline 20 weeks and follow‐up 30 weeks assessment. Sex differences in growth rate is significant: Difference = 0.12 mL/day; *t* = 4.3165; df = 844; *p* = 1.8e‐05.

Boys had significantly larger ICV at 20‐ (*B* = 2.86 ± 0.38 (s.e.); *p* = 5.7e‐14) and 30‐weeks of pregnancy (*B* = 12.35 ± 1.13 (s.e.); *p* = 8.2e‐27) (Table S4, Figure 4A–C). Moreover, boys showed more pronounced ICV growth than girls (difference = 0.12 mL/day; *t* = 4.317; *p* = 1.8e‐5) with an annualized growth rate of an average 3.15 mL vs. 3.03 mL per day (Table S4, Figure 4D).

## 4 Discussion

In this study, we present a deep learning method using a convolutional neural network for automated segmentation and measurement of fetal ICV in 3D ultrasound images. Our automated software tool is able to produce good‐quality segmentations of ICV, which seems to overcome challenges such as the fetal pose and differences in gestational age. To test the validity of our results, we investigated sex differences in fetal ICV and ICV growth in a large independent sample. We show that boys have a larger ICV at both 20 and 30 weeks of gestational age and have more pronounced ICV growth.

In the past, we have employed more traditional atlas‐based registration procedures for semi‐automated fetal brain annotations (Caspi et al. 2022). This approach consisted of a series of rigid, affine, and B‐spline refined registrations between a fetal brain image and a brain model. While being successful at quantifying the fetal brain from 3D ultrasound, we encountered several issues that we overcame in the present study. For instance, the speed in which we were now able to process the images is in the order of seconds vs. ~30–50 min per ultrasound. There are no fetal pose and age group restrictions, and our method does not require to visually QC the data before processing. That allowed us to annotate over 40,000 ultrasound images in a day rather than a few hundreds. Also, the convolutional neural network lays down a framework for segmentation of substructures of the fetal brain once sufficient multi‐label annotations are available for training in the future. This highlights the benefits of a deep learning approach over traditional image processing techniques when annotating the fetal brain from 3D ultrasound images.

Previous deep learning studies to annotate the fetal brain included varying approaches. A very deep convolutional network for large‐scale image recognition (VGG) network was developed and exploited for fetal brain detection and measurement of head circumference and biparietal diameter with an ellipse model fitted to the segmentation respectively (Simonyan and Zisserman 2014; Sinclair et al. 2018a). In addition to brain‐extraction, automatic zonal segmentation was also performed with a convolutional network for biomedical image segmentation (U‐Net) (Ronneberger, Fischer, and Brox 2015; Van Sloun et al. 2021). A multi‐task fully convolutional network technique was leveraged to the measurement of abdominal circumference, and a 3D fetal brain model was aligned to a canonical reference space (Sinclair et al. 2018b; Namburete et al. 2018). A 3D CNN approach worked well on accurately and reliably extracting the fetal brain regardless of the large data variations in the acquisition and the pose variation of the subject, the scale, and even partial feature‐obstruction in the 3D ultrasound images (Moser et al. 2020, 2022; Hesse et al. 2022). The performance of our ensemble network is in line with previous work by Moser et al. (Moser et al. 2020), who proposed a similar CNN for fetal brain segmentation from 3D ultrasound. Indeed, our findings strengthen the potential of CNN for fetal brain segmentation purposes. The latest advances of applying deep learning approaches in multisite fetal ultrasound datasets has led to a normative digital atlas of fetal brain maturation with automated segmentations of total brain volume, cerebellar volume, choroid plexus volume, cortical plate volume, and cortical surface area (Namburete et al. 2023). These developments allow for promising next future steps in investigating very early brain development on a large scale.

As a proof of principle, we investigated whether the automated generated annotations had the sensitivity to detect sex differences in fetal brain structure and development. To the best of our knowledge, we are the first longitudinal ultrasound study to show sex differences in fetal ICV development in 7672 ultrasound images from 1762 fetuses. Our findings are in line with previous ultrasound studies investigating in utero head circumference (as a 2D proxy for ICV), where sex differences were reported as early as 15 weeks of gestation (L'ubuský et al. 2006; Schwärzler et al. 2004; Melamed et al. 2013; Yeo et al. 2017; Broere‐Brown et al. 2016). Our findings also support and extend the well‐known finding that the median head circumference of males at birth is larger than those of females (Centers for Disease Control and Prevention 2017). Sexual dimorphisms are seen postnatally in behavior, language development and neurodevelopmental disorders (e.g., autism spectrum disorder and attention deficit hyperactivity disorder) (May et al. 2019). Boys had a larger ICV when compared to girls in utero already at 20 weeks of gestation confirming that sex differences occur already early during development. Moreover, boys when compared to girls had a more pronounced growth of ICV between 20 and 30 weeks of gestation. These findings suggest that already around 20 weeks of gestation the brains of boys and girls develop differently, and in the period between 20 and 30 weeks of pregnancy this differential growth becomes more pronounced. Interestingly, the latest work by Namburete et al. suggests that when adjusting for total brain size, sexual dimorphisms in fetal brain substructures have not been yet manifested before week 31 of gestational age (Namburete et al. 2023). Our findings further confirm that sex differences might have a very early and prenatal origin; however, in our cohort, the sex differences seem to represent a global rather than a local effect. Furthermore, our findings highlight that we were able to register well‐recognized fetal brain size differences, without including an additional manual quality control step for each of the thousands of ultrasounds we annotated. This approach opens opportunities to study the impact of prenatal brain development on postnatal outcome on a large scale.

Our model showed good overall performance in our high‐quality annotated test data; however, when applying in the large cohort data with many raw ultrasound images per person with varying quality and regions of interest, we also encountered some issues. Total brain volume is very challenging to segment in ultrasounds because of low contrast in tissue boundaries between the skull, CSF, dura and brain. Because ICV is a good proxy for brain size, it can act as a substitute marker of neurodevelopment, where deviations in ICV may suggest atypical neurodevelopment early in life, but we must acknowledge that ICV does not capture the full extent of early brain development. Only a limited amount of labeled data was available and therefore a data augmentation step was employed. Although data augmentation increases the diversity of samples in small training datasets to provide invariance against known alterations of the input data (such as various degrees of rotation, cropping, and signal‐to‐noise), there are some concerns about overfitting. The current training sample is relatively small for training complex models, and some restrictions apply, such as distribution of sex and availability of complete data across the repeated measures after quality control. In addition, the YOUth Baby and Child cohort data has a specific study design with two repeated measures between roughly 19–23 and 29–33 weeks of gestational age, instead of a continuous distribution of ages. The brain develops rapidly between these two time points, with ICV expanding three‐fold within these 10 weeks as we confirmed, and with dramatically increased gyrification (Armstrong et al. 1995). It seems that our model had the tendency to over‐annotate the smaller 20‐week brains and under‐annotate the larger 30‐week brains. As a next step, we will investigate whether we can optimize our model to better account for our longitudinal study design. In addition, in this study we took a registration‐based approach to extract the fetal brains from the ultrasound volume, which is a computationally intensive step. Adding an initial skull localization step to the segmentation procedure as proposed in Moser et al. (Moser et al. 2022) is preferred and will therefore be included in the next iteration of the software. Moreover, next steps will also include adding segmentation confidence through a Bayesian Monte Carlo dropout approach (Van Sloun et al. 2021) and the use of high contrast labeled fetal MRI to extend the pipeline beyond ICV for annotations of cortical and subcortical brain structure in ultrasounds (Khalili et al. 2019) and improve the quality control procedure.

## 5 Conclusion

In summary, we presented an automated tool for extracting fetal brain structure from 3D ultrasound, which predicted good‐quality segmentation of fetal ICV. Our automated software tool performed accurately for 3D ultrasound images acquired between 19 and 33 weeks of gestational age and provided reliable ICV results. The automated software tool provided us with the opportunity to investigate fetal brain structure on a much larger scale, and we were able to show significant sex differences in early brain development. In the future, we would extend our network by predicting multiple classifications for substructures inside the fetal brain or by multi‐task learning techniques to explore more biological problems. In a clinical setting, such automated software tools might ultimately aid in the screening for potential neurodevelopmental malformations.

## Ethics Statement

The studies involving human participants were reviewed and approved by Medical Research Ethics Committee Utrecht. Written informed consent to participate in this study was provided by the participants' legal guardian/next of kin.

## Conflicts of Interest

The authors declare no conflicts of interests.

## Supporting information


Data S1.

## Data Availability

The data that support the findings of this study are available from the corresponding author upon reasonable request.
