# Supplementary material for: Automated Segmentation of Fetal Intracranial Volume in Three‐Dimensional Ultrasound Using Deep Learning: Identifying Sex Differences in Prenatal Brain Development
Source: Hum Brain Mapp. 2024 Dec 4;45(17):e70058. doi: 10.1002/hbm.70058 (PMC11615793; doi:10.1002/hbm.70058)
Supplement: Supplementary file 1 — Data S1. [file HBM-45-e70058-s001.docx]

**Supplementary Material**

Procedure for the manual quality inspection and annotation of intracranial volume in ultrasound scans

Quality control procedure

Ultrasound scans were inspected for quality in GE’s 4DView software by experts trained to assess and annotate fetal ultrasounds. Selection criteria for the quality control were coverage of (almost) the entire brain based on the skull contour, no discernable motion or acquisition artifacts, enough signal and contrast of the skull and brain tissue to perform a reliable annotation of the intracranial volume (see description of annotation procedure below). No additional exclusion criteria beyond those of the original study criteria and the aforementioned image quality were applied. This decision to include a broad diversity of subjects and possible conditions was made to avoid potential bias of the classifier and ensure generalizability of the classifier to a population that includes birth complications.

Annotation procedure

Annotation of intracranial volume of fetal brain ultrasound scans in the training dataset was performed by an in-house tool for semi-automatic registration and segmentation of intracranial volume, originally intended for magnetic resonance imaging scans (Caspi et al. 2022). Manual inspection of the annotations were performed by experts trained to assess and annotate fetal ultrasounds by creating an overlay of the intracranial volume annotation mask on top of the original ultrasound scan.


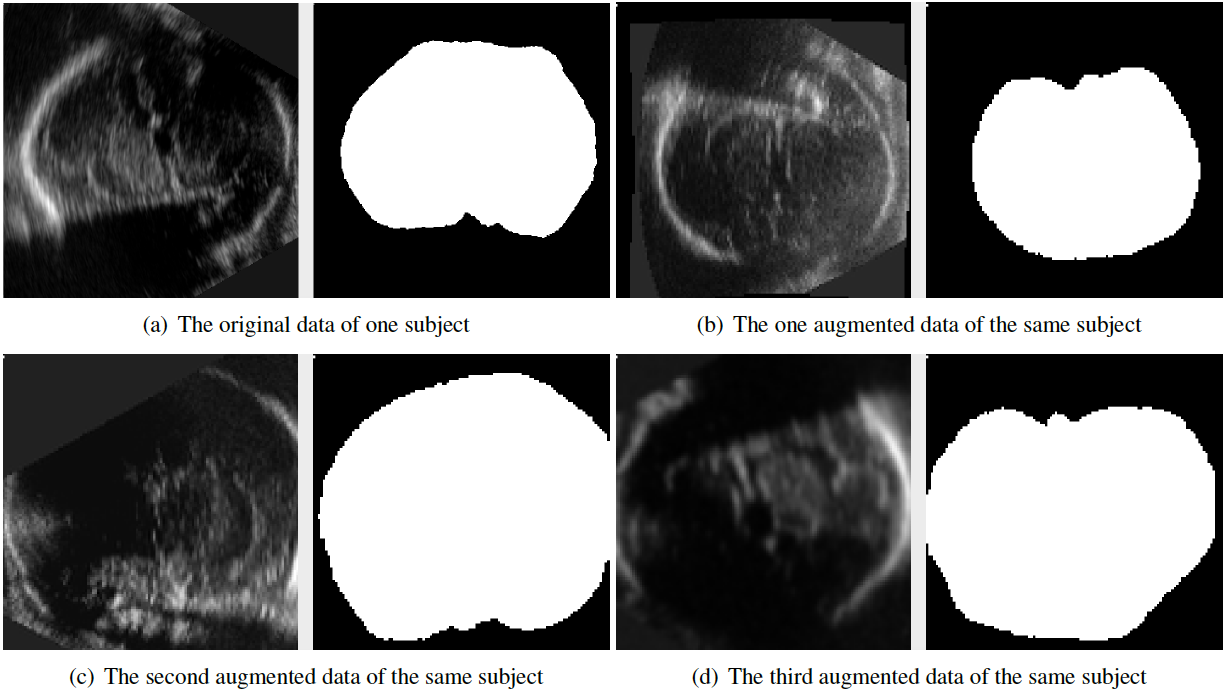

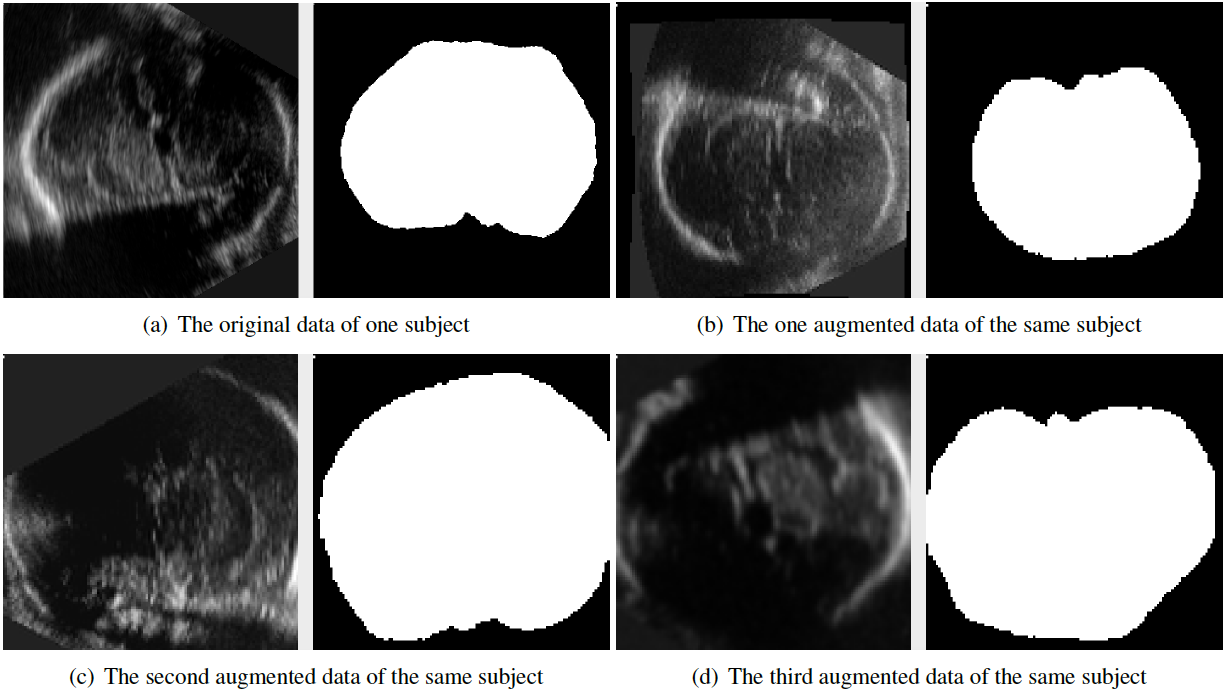


**Figure S1**: Examples of data augmentation in one participant at 30 weeks of gestation. For each subplot, there is one slice of 3D ultrasound image with corresponding 3D annotation. Top left: the original image and annotation. Top right: the augmented data included mirroring. Bottom left: the augmented data included, e.g., scaling. Bottom right: the augmented data included mirroring, scaling, blurring.

| 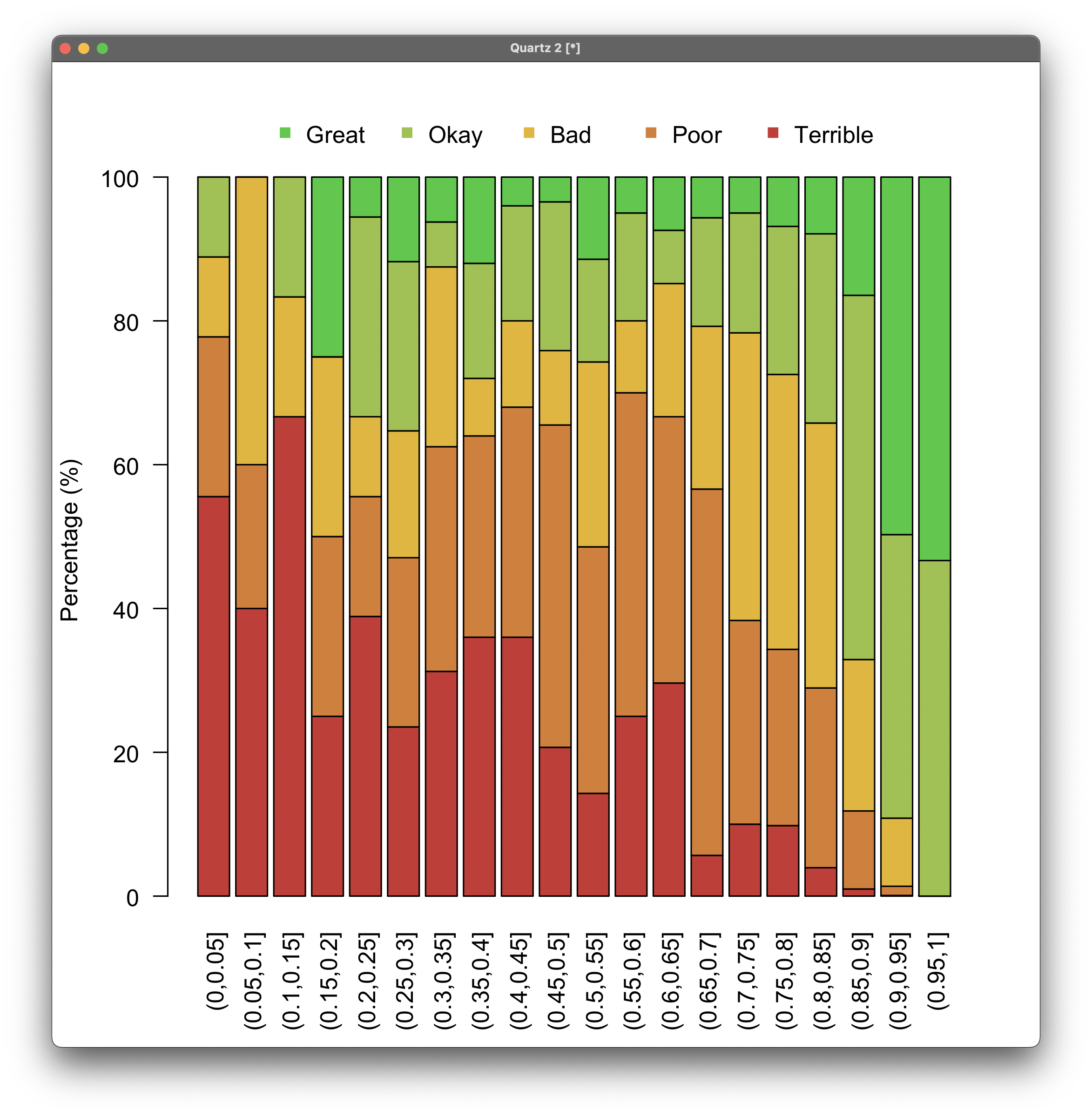 | 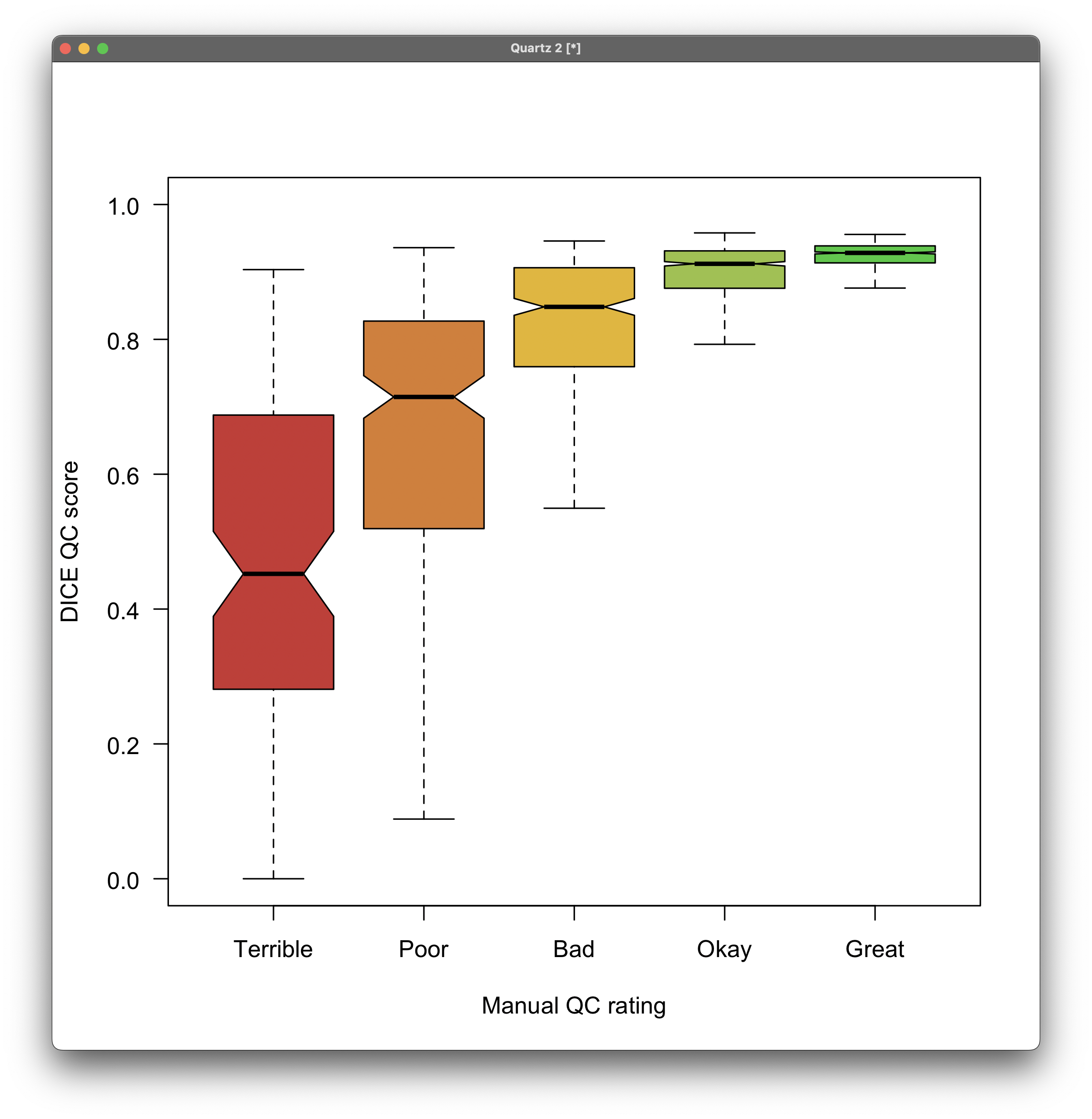 |
| --- | --- |

**Figure S2**: Validation automated DSC QC score. An expert rated the ICV masks overlayed on their ultrasound to assess the quality of the segmentation. **(A)** Percentage five quality labels per DSC QC score bin. **(B)** relationship between manual QC rating and DSC QC score.

| 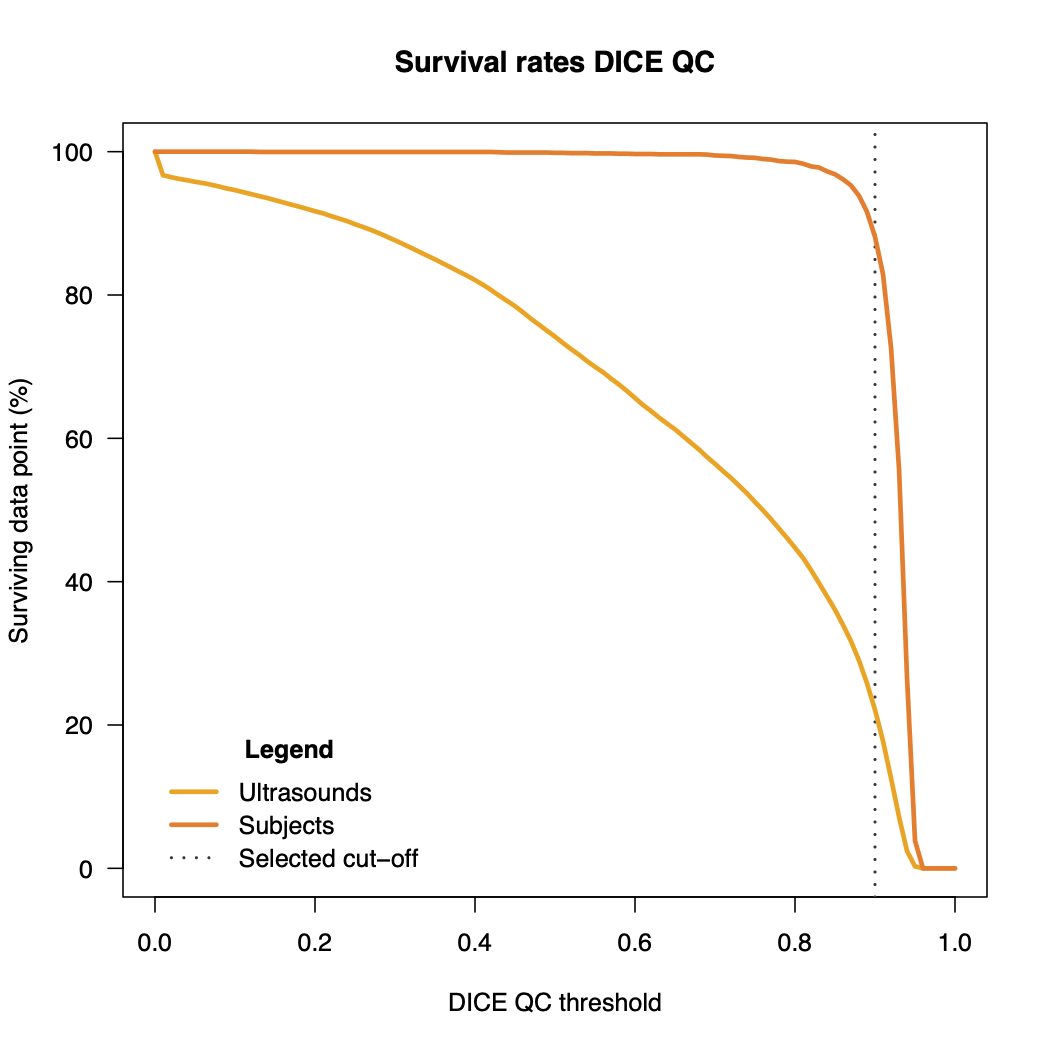 | 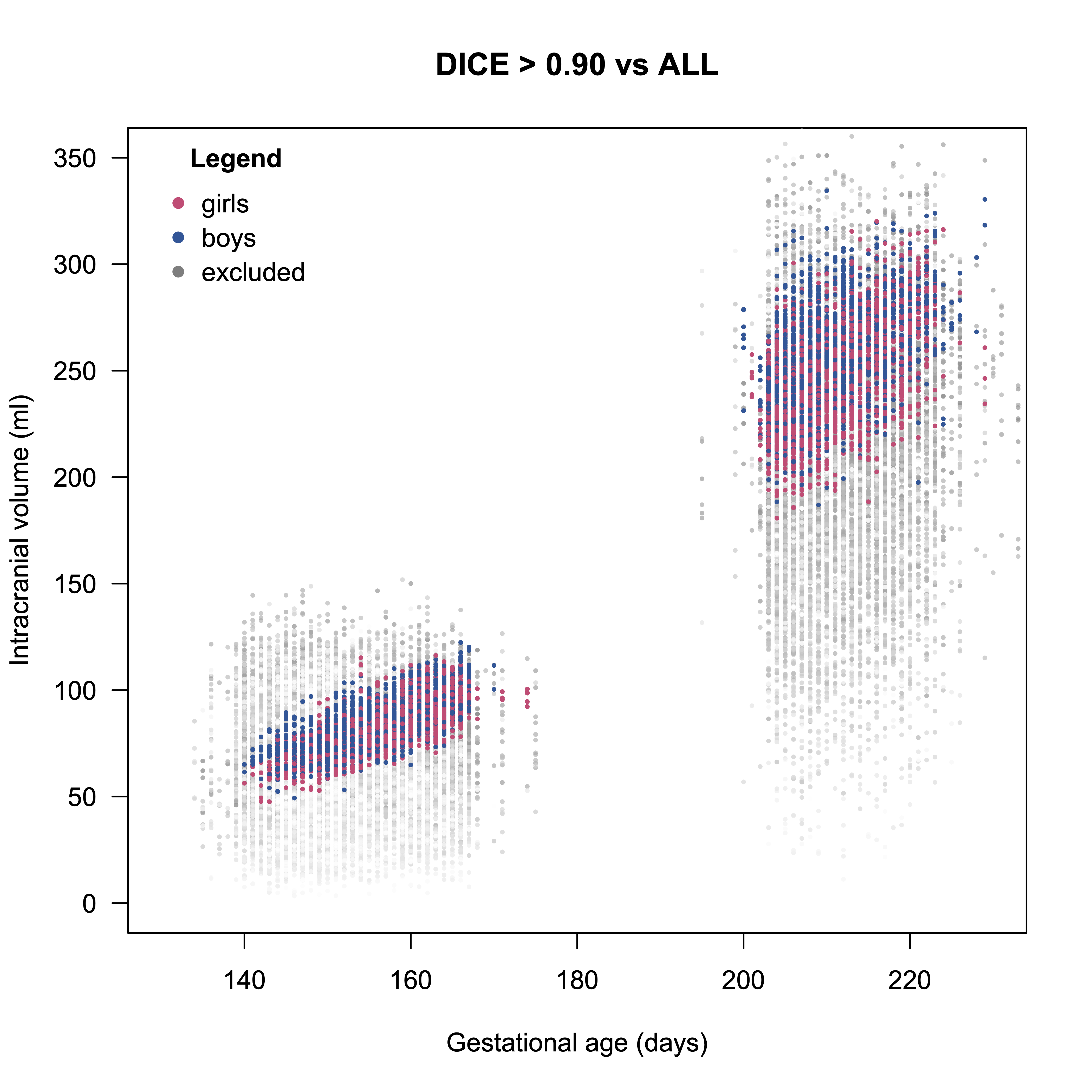 |
| --- | --- |

**Figure S3**: (**A**) Percentage of ultrasound volumes and subjects with at least one ultrasound volume that remain at different cut-off threshold for the DICE QC score, with a mark at the selected cut-off threshold DICE QC score > 0.90 used. (**B**) The ICV volumes of ultrasounds with a DSC > 0.90 included in the statistical analyses compared to the volumes of all available ultrasounds (colored=included; gray=excluded).

Convergence of model parameters

The models with the best performance in each cross fold (network #5, #13, #17) were inspected for convergence of model parameters (Figure S4). All three networks show a good fit between train data and validation data: the voxel-wise accuracy and DSC spatial overlap started to converge around the 5th epoch and steadily increased afterward. The loss function of the three networks decreased rapidly in the first 5 epochs before steadily declining further in the remaining epochs. In order to obtain a powerful and consistent model, we combined the three networks as an ensemble network with a voting scheme to reduce the impact of variation between new datasets and improve the quality of segmentation.


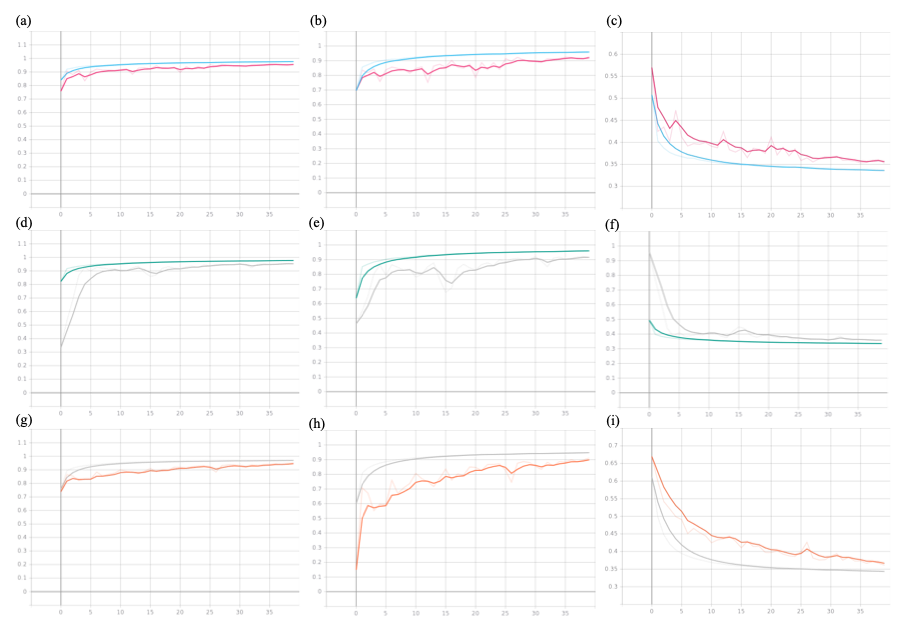


**Figure S4.** The performance of the best three networks; No.5 (a, b, c), No.13 (d, e, f) and No.17 (g, h, i) over 40 epochs. The left column depicts the voxel-wise accuracy dynamic, the middle column for the Dice Similarity Coefficient (DSC) dynamic and the right column for the Sparse Categorical Cross-Entropy loss dynamic measuring the similarity/difference between annotation and prediction data during training. In each subplot, the two curves represent the train data (blue, green, gray) and validation data (purple, gray, orange) respectively.


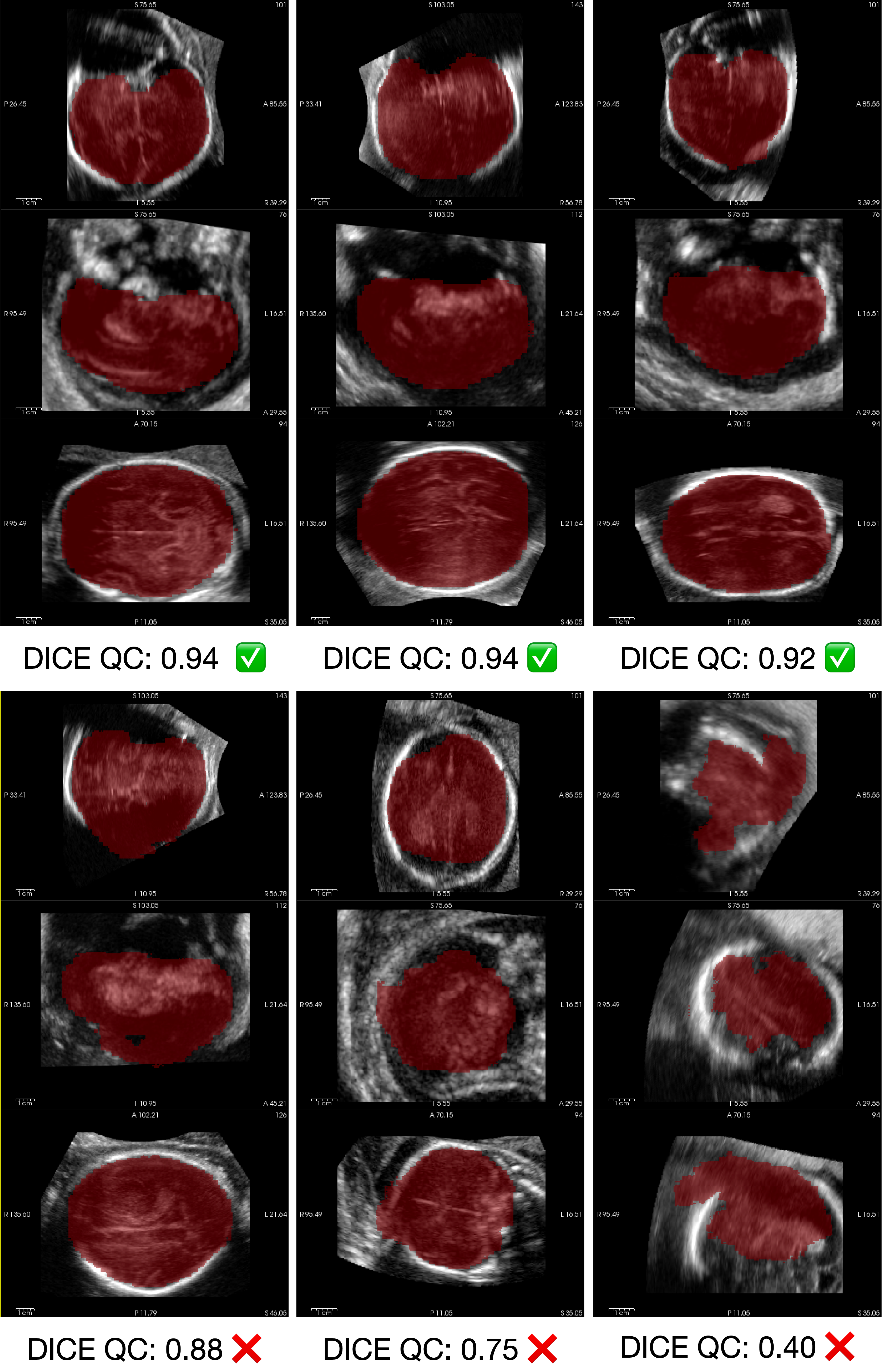


**Figure S5.** Examples of ICV masks overlayed on top of corresponding ultrasound and DSC QC score. Top row: three accepted segmentations, two examples with very good quality and one example just above the DSC QC threshold (with slight overfitting at the top of the skull). Bottom row: three rejected segmentations, one example (left) just below the DSC QC threshold with overfitting, one example (middle) clearly failed to properly segment the ICV, and one example (right) with a bad ultrasound (and therefore also with a bad segmentation).

**Table S1.** Sample characterics of participants included in the train, validation and test set (N=95)

|  | N | Mean | SD | Min | Max |
| --- | --- | --- | --- | --- | --- |
| Birthweight (grams) | 81 | 3492.31 | 477.17 | 2404 | 4810 |
| Apgar score (1 min) | 75 | 8.85 | 1.07 | 3 | 10 |
| Apgar score (5 min) | 76 | 9.72 | 0.58 | 7 | 10 |
| Maternal age | 95 | 32.27 | 3.97 | 20 | 42 |
| BMI mother | 92 | 24.12 | 3.97 | 16.90 | 38.82 |

|  | Y/N | % yes |
| --- | --- | --- |
| Smoking (pre-pregnancy)^a^ | 18/65 | 21.7% |
| Smoking (pregnancy 20w)^a^ | 1/82 | 1.2% |
| Alcohol consumption (pre-pregnancy)^b^ | 52/31 | 62.7% |
| Alcohol consumption (pregnancy 20w)^b^ | 24/59 | 28.9% |

| Monthly household income^c^ | <€1,250: 1 (1.1%)  €1,250 to €2,000: 3 (3.3%)  €2,000 to €3,000: 5 (5.4%)  €3,000 to €4,000: 19 (21%)  >€4,000: 60 (65%)  Undisclosed: 3 (3.3%)  Unknown: 1 (1.1%) |
| --- | --- |
| Maternal education level^d^ | Voortgezet onderwijs of lager: 5 (5.4%)  Middelbaar beroepsonderwijs (MBO): 18 (20%)  Hoger beroepsonderwijs (HBO): 18 (20%)  Wetenschappelijk onderwijs (WO): 51 (55%) |
| Maternal marital status | Married: 43 (47%)  Never married: 38 (41%)  Divorced: 1 (1.1%)  Widow: 1 (1.1%)  Registered partnership: 9 (9.8%) |

1. Measured as any kind of tobacco smoking
2. Alcohol consumption was measured as one or more glasses of alcohol a week.
3. Monthly household income is reported for maternal household only if not married or living together with partner, or combined income if married and/or living together
4. Maternal education level was measured as the highest completed degree of the Dutch education system. “Voortgezet onderwijs of lager”: mandatory education up to age 16–18 years (equivalent to high school or secondary education), for up to 14 years of education, depending on education tract; “Middelbaar beroepsonderwijs (MBO)”: secondary vocational education, for an additional 4 years of education; “Hoger beroepsonderwijs (HBO)”: higher professional education, for an additional 4–5 years of education; “Wetenschappelijk onderwijs (WO)”; university degree, for an additional 3–5 years of education); see also <https://en.wikipedia.org/wiki/Education_in_the_Netherlands>.

**Table S2.** Sample characterics of participants included in the main analyses (N=1762)

|  | N | Mean | SD | Min | Max |
| --- | --- | --- | --- | --- | --- |
| Birthweight (grams) | 1459 | 3520.00 | 499.56 | 480 | 6165 |
| Apgar score (1 min) | 1394 | 8.74 | 1.31 | 0 | 10 |
| Apgar score (5 min) | 1397 | 9.67 | 0.91 | 0 | 10 |
| Maternal age | 1675 | 32.19 | 3.80 | 20 | 50 |
| BMI mother | 1662 | 23.55 | 3.87 | 15.82 | 44.92 |

|  | Y/N | % yes |
| --- | --- | --- |
| Smoking (pre-pregnancy)^a^ | 175/1451 | 10.8% |
| Smoking (pregnancy 20w)^a^ | 32/1594 | 2.0% |
| Alcohol consumption (pre-pregnancy)^b^ | 907/718 | 55.8% |
| Alcohol consumption (pregnancy 20w)^b^ | 364/1249 | 22.6% |

| Monthly household income | <€1,250: 11 (0.7%)  €1,250 to €2,000: 31 (1.9%)  €2,000 to €3,000: 101 (6.1%)  €3,000 to €4,000: 271 (16%)  >€4,000: 1160 (70%)  Undisclosed: 61 (3.7%)  Unknown: 22 (1.3%) |
| --- | --- |
| Maternal education level | Voortgezet onderwijs of lager = 90 (5.4%)  Middelbaar beroepsonderwijs (MBO): 205 (12%)  Hoger beroepsonderwijs (HBO): 463 (28%)  Wetenschappelijk onderwijs (WO): 906 (54%) |
| Maternal marital status | Married: 736 (44%)  Never married: 701 (42%)  Divorced: 28 (1.7%)  Widow: 1 (0.1%)  Registered partnership: 192 (12%) |

1. Measured as any kind of tobacco smoking
2. Alcohol consumption was measured as one or more glasses of alcohol a week.
3. Monthly household income is reported for maternal household only if not married or living together with partner, or combined income if married and/or living together
4. Maternal education level was measured as the highest completed degree of the Dutch education system. “Voortgezet onderwijs of lager”: mandatory education up to age 16–18 years (equivalent to high school or secondary education), for up to 14 years of education, depending on education tract; “Middelbaar beroepsonderwijs (MBO)”: secondary vocational education, for an additional 4 years of education; “Hoger beroepsonderwijs (HBO)”: higher professional education, for an additional 4–5 years of education; “Wetenschappelijk onderwijs (WO)”; university degree, for an additional 3–5 years of education); see also <https://en.wikipedia.org/wiki/Education_in_the_Netherlands>.

Selection of hyperparameters ranges

The range of hyperparameters for filter size, batch size, and learning rate for model fitting were determined by initial trial runs where hyperparameters were divided into two groups for batch size and three groups for filter size and learning rate by the constraints commonly used by others (Table S3). The set of hyperparameters with filter size ranging from 16 to 32, batch size ranging from 16 to 32, and learning rate ranging from 0.001 to 0.005 accounted for almost one-third of the number of networks across three validation data and produced one of the highest average voxel-wise accuracy of 0.95 and highest average spatial overlap with a DSC of 0.90, which suggested that these ranges of hyperparameters can achieve good and consistent outcomes regardless of the different datasets. Although it should be noted that other ranges of learning rate had little impact on the performance, other ranges of filter size and batch size had more detrimental impact on the performance, in particular when both ranges were altered, but regardless still remained high (lowest accuracy of 0.88, and lowest spatial overlap of 0.76).

**Table S3. Optimal Hyperparameter selection.** The pre-defined filter size ranged from 8 to 64 divided into three groups, batch size ranged from 16 to 64 divided into two groups, and learning rate ranged from 0.0001 to 0.01 divided into three groups. The square bracket means the closed interval and the round bracket is the open interval. Number of networks with hyperparameters in this range. The average voxel-wise accuracy and Dice Similarity Coefficient (DSC) between networks.

| **Filter size range** | **Batch size range** | **Learning rate range** | **Network count** | **Average voxel-wise accuracy** | **Average spatial overlap** |
| --- | --- | --- | --- | --- | --- |
| [8, 16) | [32, 64] | [.0001, .0010) | 2 | 0.88 | 0.76 |
| [16, 32) | [16, 32) | [.0001, .0010) | 2 | 0.95 | 0.91 |
| **[16, 32)** | **[16, 32)** | **[.0010, .0050)** | **9** | **0.95** | **0.90** |
| [16, 32) | [16, 32) | [.0050, .0100] | 3 | 0.94 | 0.89 |
| [16, 32) | [32, 64] | [.0050, .0100] | 1 | 0.93 | 0.87 |
| [32, 64] | [16, 32) | [.0001, .0010) | 1 | 0.95 | 0.92 |
| [32, 64] | [32, 64] | [.0001, .0010) | 3 | 0.90 | 0.81 |
| [32, 64] | [32, 64] | [.0010, .0050) | 2 | 0.92 | 0.85 |
| [32, 64] | [32, 64] | [.0050, .0100] | 1 | 0.92 | 0.86 |

**Table S4**. **Fetal intracranial volume growth and sex differences**. Regression coefficients of sex and linear- and quadratic age effects on fetal ICV in milliliter (ml) at baseline around 20 weeks and follow-up around 30 weeks. The B-coefficients and standard error (SE) are reported together with nominal *p*-values. A positive sex effect indicates larger ICV volumes for boys compared to girls. Longitudinal growth rates in ml/day were computed by the difference in ICV volume at 30 weeks follow-up subtracted by the ICV volume at 20 weeks baseline assessment.

|  | **Baseline**  **20 weeks** | **Follow-up**  **30 weeks** | **Longitudinal**  **growth rate** |
| --- | --- | --- | --- |
| Intercept | 149.91±112.10 | -1,633.15±746.07 | N/A |
| Sex | 2.86±0.38  ***p*=5.6e-14** | 12.35±1.13  ***p*=8.2e-27** | t = 4.317; df = 844  ***p*=1.8e-05** |
| Age | -2.36±1.45  *p*=0.102 | 15.59±6.71  ***p*=0.026** | N/A |
| Age^2 | 1.23e-02±4.66e-03  ***p*=0.008** | -3.17e-02±1.65e–02  *p*=0.055 | N/A |
